# Special Issue: “Latest Advances on Urinary Incontinence”

**DOI:** 10.3390/jcm12227075

**Published:** 2023-11-14

**Authors:** Riccardo Bientinesi, Filippo Gavi, Vincenzo Li Marzi, Emilio Sacco

**Affiliations:** 1 Department of Urology, Fondazione Policlinico Universitario A. Gemelli IRCCS, Università Cattolica del Sacro Cuore, 00168 Rome, Italy; riccardo.bientinesi@gmail.com (R.B.); emiliosacco@gmail.com (E.S.); 2 Unit of Urological Robotic Surgery and Renal Transplantation, Azienda Ospedaliera Careggi, 50134 Florence, Italy; limarzi2012@gmail.com

Urinary incontinence (UI) has a great impact on patients’ quality of life [1,2]. In recent years, efforts have been made to develop a better and minimally invasive procedure to treat UI [3,4,5]. In this Special Issue, experts on functional and neuro-urology gave their contribution providing new solid scientific evidence in the diagnosis and management of UI.

Patients with neurological conditions usually present filling and voiding vesical disfunctions [6]. Some of these patients are treated with intermittent catheterizations or permanent vesical catheters. Catheter-associated infections (CAUTI) are a burden in hospitals and long-term care settings. Musco et al. [7] published a review of the literature focusing on the strategies that we can adopt to reduce CAUTI. Bientinesi et al. [8] provided a comprehensive review of the literature describing the impact of neurogenic urinary incontinence (nUI), highlighting the treatment options and the roles that each specialist has in the management of these complex patients. Moreover, in a single cohort study, nUI and sexual dysfunctions were shown to have a great impact on the QoL of patients with multiple sclerosis being a major issue to be addressed [9]. Jaekel et al. [10] reported the burden that nUI has on patients and their caregivers. Abidi et al. [11] published a paper demonstrating that UI is associated with worse physical outcomes.

UI is one of the most frequent complications after robot-assisted radical prostatectomy (RARP) [12,13]. RARP can be performed with several techniques and different robotic platforms, constant efforts are made to improve functional outcomes [14,15,16]. Gacci et al. [17] published a review with the latest evidence on UI after RARP. Sessa et al. [18] conducted a prospective study reporting the results after RARP at their center showing that anterior and/or posterior fascial reconstruction might improve early incontinence rate. Artificial urinary sphincter (AUS) is still the gold standard in the treatment of UI after RARP, but complications are possible. Sacco et al. [19] published a paper on a refined technique that decreases significantly surgical complications. Geretto et al. [20] compared AUS and adjustable male sling positioning reporting a higher continence success rate in the AUS group although, sling can be a better alternative in patients requiring a less invasive procedure. Chiu et al. [21] conducted a study on the efficacy and safety of sling positioning, reporting that even in the severe UI group some patients had good continence after surgery.

Women present a high prevalence of UI. Prevalence in middle-aged and post-menopausal women can be as high as 50% [22]. Giammò et al. [23] published the results of a multicentric study on the use of urethral bulking in the treatment of non-neurogenic female stress and mixed urinary incontinence. Women with mild UI seem to be the ones to benefit more from this treatment. Venema et al. [24] published their opinion on the role of urethral smooth musculature during normal bladder filling in women. In the treatment of UI in women, magnetic stimulation may be one of the available options as Braga et al. suggested [25]. 

Overactive bladder (OAB) can be a great burden on patients and treatments are often not effective or not tolerated [26]. It is, therefore, important to inform patients and to understand their expectations that sometimes can be too high [27]. Cicione et al. [28] published a systematic review focusing on patients’ preferences and expectations reporting that the ideal medication should be cheap, without risk of cognitive function impairment, and able to reduce daytime urinary frequency and incontinence episodes. Several treatments can be proposed, among them physical and agent-based treatment can be offered to a selective group of patients [29]. 

In conclusion, this Special Issue covers optimal treatments and minimally invasive procedures highlighting important new results in the field. The papers listed here are extremely helpful additions that address knowledge gaps in several areas related to UI. We believe that the authors’ results will be a useful resource for researchers and an inspiration for upcoming research.
